# Multi-Angle Averaging Approach for Measuring the Coating Thickness on Thin Transparent Polymer Films

**DOI:** 10.1177/00037028251334152

**Published:** 2025-05-14

**Authors:** Friederike Münch, Benedikt Hauer, Ingo Breunig, Daniel Carl

**Affiliations:** 1 28468 Fraunhofer Institute for Physical Measurement Techniques IPM, Freiburg, Germany; 2 Department of Microsystems Engineering—IMTEK, University of Freiburg, Freiburg, Germany; 3 Department of Sustainable Systems Engineering—INATECH, University of Freiburg, Freiburg, Germany

**Keywords:** Thin films, polymer web, infrared spectroscopy, IR‌, silicon oxide, inline thickness measurement, roll-to-roll coating systems, production control

## Abstract

Polymer films with a thickness in the two-digit micrometer range are coated with nanometer-thin oxide layers in roll-to-roll coating systems. The coating improves the properties of the film, such as gas or water permeation. Maintaining a sufficiently large coating thickness is crucial to ensure its barrier function; thus, inline quality control of the thickness is indispensable. For this purpose, we have developed a sensing principle that addresses specific absorption bands of the coating via a reflection measurement in the infrared spectral range. However, for thin and weakly absorbing polymer substrates, light is reflected not only by the coating and the surface of the polymer. Partly it is also transmitted and reflected by the backside of the film, leading to interference effects that significantly affect the measurement signal. As industrial films vary in thickness by several percent and their exact values are unknown, determining the thickness of an oxide coating is hindered. In this paper, we demonstrate an approach for measuring coating thickness on such varying polymer films by averaging the interferences obtained at multiple angles of incidence. Calculations and measurements on industrial film samples indicate the effectiveness of our approach. It produces results with ±2 nm precision and ±5 nm accuracy for a thickness in the range of 5–100 nm. Furthermore, we discuss a possible implementation of this approach in an inline measurement system by fulfilling its requirements, for example, versatility and compactness.

## Graphical abstract

**Graphical abstract:**
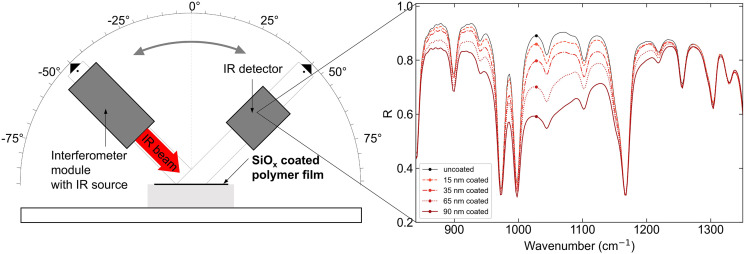
This is a visual representation of the abstract.

## Introduction

Polymers such as polypropylene (PP) and polyethylene (PE) are cost-efficient and suitable for mass products such as food and pharmaceutical packaging or flexible electronic devices. In this context, applying an oxide coating to the polymers changes and enhances their properties, for example, a significant reduction in gas permeation (e.g., O_2_ and CO_2_) and vapors (e.g., H_2_O and aromas) or electrical insulation. Furthermore, the polymers can be manufactured as thin, flexible films, allowing for serial coating production using chemical or physical vapor deposition methods in roll-to-roll (R2R) coating systems.^1,2^

As coating is important for product properties, inline control is essential to ensure its quality and optimize the coating process. One critical parameter in this context is the thickness of the coating. Maintaining a sufficiently thick coating layer is crucial for achieving a fully cross-linked coating structure with its barrier function. However, the coating should also not be applied too thickly to avoid wasting resources. For the widely used silicon oxide (SiO ${}_{x}$ ) coatings discussed here, a thickness of minimum 10nm is required.^3^

For inline quality control, the measurement system must be non-destructive, capable of rapid process-adapted measurements, compact, and versatile for various scenarios and materials.^4^ However, such systems are not yet widely used in R2R systems, although there are already approaches for inline applications.

Yersak et al.^5^ demonstrate a laboratory-scale inline system that measures the thickness of an aluminum oxide coating on a moving PE naphthalate (PEN) web using reflectometry in the ultraviolet (UV) spectral range. They reach an accuracy of ±2nm for a 50nm coating layer. This method is suitable for coating thicknesses of the order of 30nm and more, but becomes less accurate when resolving thinner or highly transparent layers.

Similarly, Logothetidis et al.^6^ demonstrated an inline measurement system for lab-scale R2R coating systems based on ellipsometry. They measure, for example, a 70nm SiO ${}_{x}$ layer on a 50μm thick PE terephthalate (PET) substrate. The effectiveness of this method is based on the optical contrast between the coating and the substrate material. The contrast is sufficiently high in the absorption bands of the substrate or the coating material. For instance, Logothetidis et al. utilize the absorption of PET in the UV region and reach a precision of ±1nm .

X-ray fluorescence (XRF) is a versatile method used in industrial R2R coating systems, for example, for measurements of film thicknesses.^7,8^ Nanometer-thin oxide coatings, such as SiO ${}_{x}$ , on polymer films are measurable.^9^ However, XRF systems are relatively slow, with measurement times mainly in the range of several seconds and cost-intensive.^8^

In addition to the measurement approaches for films in R2R systems, inline inspection systems are also used for batch production. These involve measuring the film thickness via thin film reflection and absorption within the infrared (IR) spectral range.

An example is to inspect the thickness of the coating in molded containers.^10^ The thickness of the nanometer-thin oxide coatings is measured through their absorption bands in the IR range. With a similar method thicknesses down to 5nm are measurable, as demonstrated by Hauer et al.^11^ This method does not rely on spectroscopy. Instead, the measured reflectance within the spectral position of the absorption band is correlated to the thickness of the coating. A quantitative measurement therefore requires proper calibration of the thickness-dependent reflectance.

All mentioned techniques except XRF exploit the effect that a portion of the light is reflected at the interface of the air and the coating and at the interface of the coating and the substrate.

However, with even thinner substrates, typically in the range of 10–20 μm ,^1^ or when using less absorbing polymers such as PP, light is not fully absorbed by the polymer substrate. Reflected light waves from the back side of the substrate can cause unwanted interference with light waves reflected from the coating interfaces. An exception is within the UV-B and UV-C range, where polymers strongly absorb light, and light is fully attenuated even with thin substrates. In industrial applications, the thickness of the films fluctuates by around 5%, and its exact value is unknown. Consequently, the phase shift of the interfering light waves varies, leading to a change in the resulting intensity. This effect makes it impossible to accurately determine the change of intensity of the reflected light due to a varying coating thickness at a given spectral position without the use of simulation models and software^12,13^ to fit the measured spectrum to a theoretical model. However, it is not possible to directly determine the coating thickness from the measurement signal at a specific wavelength associated with the spectral feature of the coating.

We show a multi-angle measurement approach that averages out the disturbing interferences caused by the substrate. Measurements are taken at several angles of incidence and averaged. Thus, the mean intensity of the reflected light remains constant for slight variations of the substrate thickness. We demonstrate measurements of the coating thickness using the IR region. The oxides have strong absorption bands within this range, which makes it possible to determine thin coating layers down to the required thickness of 10nm . Furthermore, IR light is less sensitive to surface roughness and slight variation in stoichiometry than UV light.

In the following sections, we demonstrate the effectiveness of this approach based on calculations and measurements of industrial film samples. We also discuss solutions and options for implementing the approach in a measurement system and fulfilling the requirements for inline controls.

## Fundamentals

**Figure 1.**
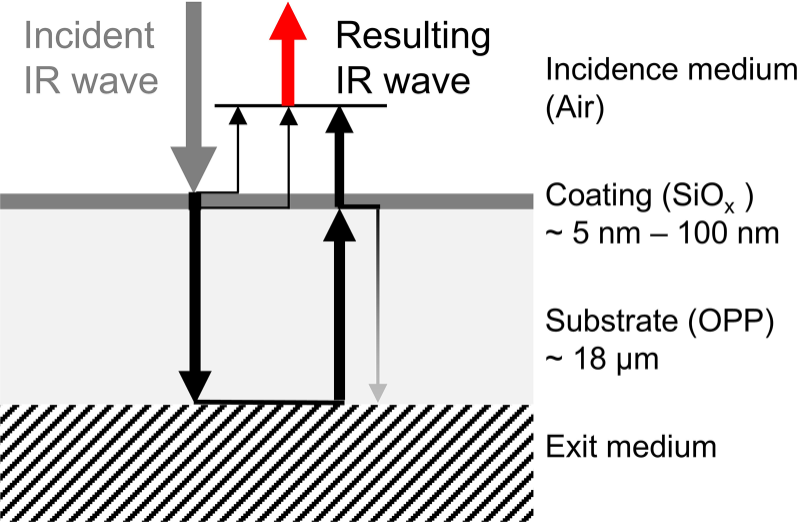
Scheme of the layer stack and the first reflected and transmitted light waves.

In order to understand the principle of the coating thickness measurement, we need to describe the characteristics and terminology of our film system. The coated polymer film, a layer stack consisting of a thin film coating layer of SiO ${}_{x}$ and a polymer substrate of oriented PP (OPP), is sketched in Figure 1. The coating layer has a thickness in the double-digit nanometer range and the OPP substrate in the double-digit micrometer range. IR light from a semi-infinite incidence medium, specifically air, hits the layer stack. The spectral range of the IR light is set in a range that addresses the Si–O vibrational band. The significant measurement value is the intensity of the reflected light from the coated polymer within this range. At each interface, the IR light is partially reflected and transmitted. However, for thin and weakly absorbing polymer layers, a portion of the light is transmitted into the semi-infinite exit medium (see Figure 1).

Firstly, to explain the measurement principle of the coating thickness, we assume that reflections do not occur at the interface of the substrate/exit medium and that light is completely absorbed by the polymer. Thus, light is only reflected at the interfaces between the semi-infinite incidence medium (air)/coating layer and the coating/substrate layer, leading to multiple reflections inside the coating and interferences. In addition, IR light is absorbed as it passes through the coating layer. As the thickness variations of the coating layer are around three orders of magnitude smaller than the wavelength, we expect that light absorption primarily affects the change in intensity. For SiO ${}_{x}$ on a polymer substrate, the highest light absorption within the coating layer is in the range of 900 – $1200\;{\text{cm}}^{-1}$ wavenumbers. In this spectral range, we measure the thickness of the coating.^14^ Under this assumption, the layer thickness could be measured directly via the intensity of the reflected light.^11^ On dielectric substrates, such as polymers, the reflectivity of s-polarized light increases with the angle of incidence, while for p-polarized light, the reflectivity decreases after reaching Brewster’s angle. Since the reflectivity of s-polarized light is significantly higher, the overall reflectivity for light without specific polarization also increases.^15^ However, the difference in reflectivity between a coated polymer and an uncoated film decreases as the angle of incidence increases. Thus, the optimal angle of incidence is a compromise between reflectivity and change in signal, covering a more extensive range of angles.

However, we now consider the case where a significant portion of light is reflected at the interface of the polymer substrate and the exit medium. This is the case for thin polymer layers with weak absorption, such as OPP with a thickness of around 18μm . The thickness of the substrate influences the measurement signal, hindering the direct determination of the coating thickness based on the intensity of the reflected light. We will explain the influence of variations in the substrate thickness on the measurement signal in the following. Derived from calculation models, we will also show how the material of the exit medium affects the resulting spectral intensity. Furthermore, we will explain the multi-angle averaging approach and demonstrate its effectiveness based on calculations.

### Influence of the Substrate Thickness

Figure 1 shows the reflections of the IR waves at the interfaces between air and coating, coating and polymer, and polymer and semi-infinite exit medium. Only the first reflections of the infinite series are sketched. All reflected IR waves interfere, marked as the resulting IR wave. The phase shifts of the light waves directly reflected at the coating interfaces are unaffected by the thickness of the substrate, but the phase shifts of the backside reflected waves are affected. A variation in the thickness of the substrate changes the phase relations. Consequently, the resulting light intensity depends also on the thickness of the substrate.

### Influence of the Semi-Infinite Exit Medium

As the substrate is optically transparent, the complex refractive index of the semi-infinite exit medium greatly influences the reflection of light.

For air as the exit medium, the reflectance at the polymer/air interface is several percent (based on Fresnel equations) and most of the light is transmitted into the exit medium. In contrast, with semi-infinite exit medium materials such as aluminum, gold, or silver, light is virtually not transmitted at the interface and the reflectance is close to 100%. As explained above, reflections from the interface of the substrate and the exit medium affect the measurement signal. It might be assumed that it would be advantageous if the intensity of the light reflected from this interface were low, as is the case with the air medium. However, the measurements are improved when the exit medium consists of metal, as explained in the following.^16,17^

If the reflectance of the polymer/exit medium interface is weak, most of the light is transmitted into the exit medium, and in a first approximation, only the first reflection at this interface has to be considered (see Figure 1). The intensities of the first reflected light waves from the interfaces of the coating and the substrate are of the same order of magnitude as the intensity of the reflected light wave from the interface of the polymer and semi-infinite exit medium. Oscillations of the light intensity over the spectral range are observable. For destructive interference, the resulting intensity is close to zero.

With semi-infinite exit medium materials with high reflectance, such as metals, the waves are entirely reflected in a first approximation at the interface of the substrate and the background material. As a result, the overall intensity is much higher. A reduction of the reflectance correlates to absorption and interferences within the layers. Furthermore, the phase of the light wave reflected at the polymer/metal interface shifts by approximately $180^{\circ }$ , which contributes to partial destructive interference, resulting in less pronounced interference fringes.^17^

However, multiple reflections with non-negligible intensity occur in the polymer and the coating layer. For example, light waves reflected from the interface of the polymer and exit medium are partially reflected and transmitted at the polymer/coating interface.

The multiple reflections with a significant intensity lead to a more pronounced effect of the absorption by the coating layer in the overall reflectance than in the case where the exit medium is air.

### Reflectance of the Layer Stack

Due to the multiple reflections, the intensity of the resulting light wave cannot be efficiently determined by considering individual reflections at the interfaces. Thus, its reflectance spectra are calculated using the transfer matrix method (TMM), considering all multiple reflections in the layers.^14,15,18^ For calculating the reflectance over the IR spectral range, we use the Python software package provided by Byrnes.^19^ Input parameters are the thickness of the layers, the complex refractive index as a function of the wavenumber of each material, and the angle of incidence.

**Figure 2.**
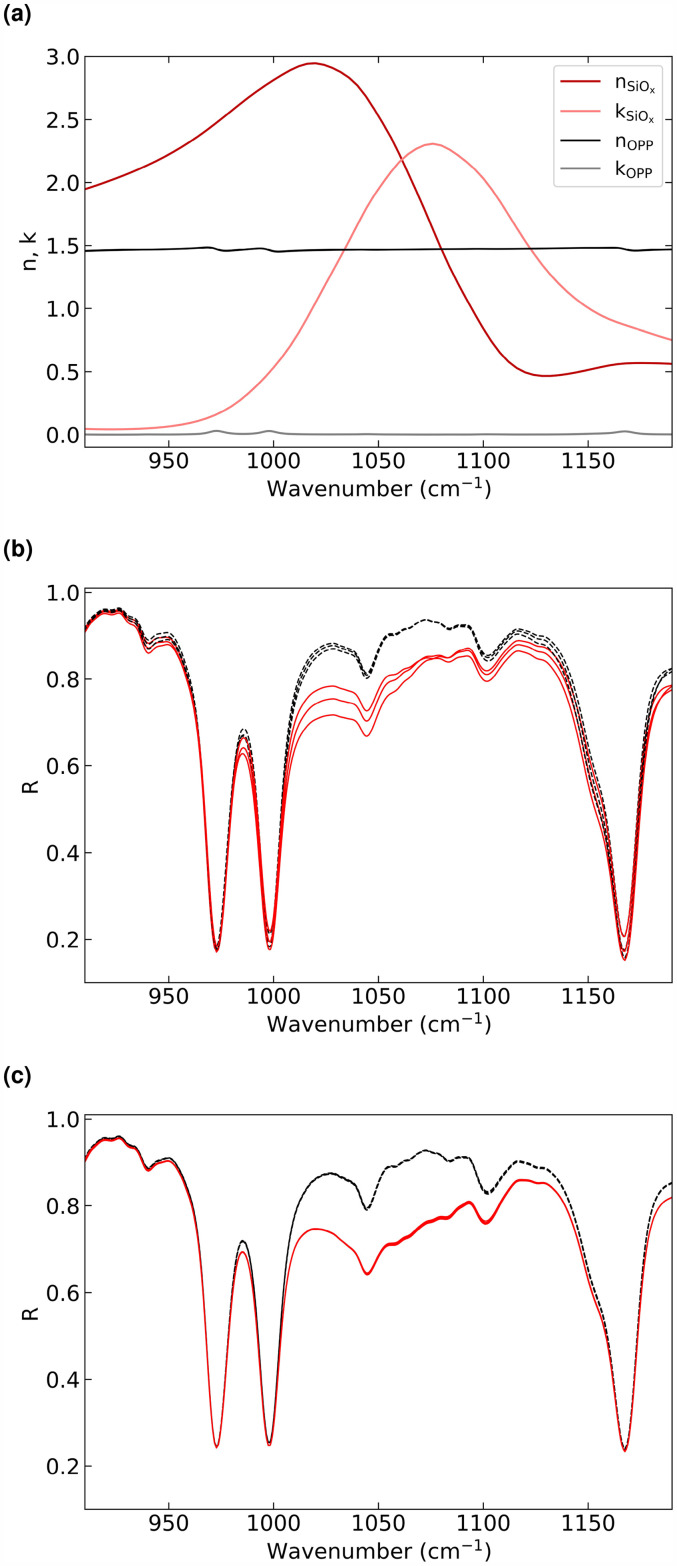
Panel (a) displays the dielectric function of SiO ${}_{x}$ ^21^ and the polymer OPP. Panels (b) and (c) show the calculated spectra for uncoated polymer films (dashed lines) and 25nm thick-coated polymer films (solid lines) with variations of the substrate thickness of 5%. Values of the substrate thickness are 17.75, 18.0, and 18.25 μm . Spectra of panel (b) are calculated with $45^{\circ }$ angle of incidence. Panel (c) shows the same calculations as in panel (b) but with the multi-angle averaging approach.

Figure 2b displays the calculated overall resulting reflectance for different substrate thicknesses of 17.75, 18.0, and 18.25 μm for uncoated polymers (dashed lines) and (SiO ${}_{x}$ ) coated polymers (solid lines). The coating thicknesses is 25nm . The calculated curves are plotted over the spectral range from around 900–1200 ${\text{cm}}^{-1}$ wavenumbers, covering the Si–O stretching, where the thickness of the coating thickness will be determined, as described above. Aluminum is used as an exit medium with high reflectance and implemented with its function of the complex refractive index from Rakić et al.^20^

The values for the function of the complex refractive index of the SiO ${}_{x}$ coating are taken from Kischkat et al.^21^ The function is shown in Figure 2b, n refers to the real part and k to the imaginary part.

The complex refractive index function of the polymer OPP (shown in Figure 2a) was determined from Fourier transform infrared spectroscopy (FT-IR) reflection and transmission measurements near normal incidence ( $∼\;30^{\circ }$ ) on an OPP film placed on an aluminum mirror. For determining the function of the complex refractive index, we use the software RefFIT by fitting a series of (Kramers–Kronig consistent) oscillators as described by Kuzmenko.^22^ We used these functions to calculate the spectra via the TMM.

The angle of incidence is set to $45^{\circ }$ and represents a compromise between the intensity of the reflected light and the change in signal.

Figure 2b shows that the intensities of the coated samples are reduced compared to those of the uncoated samples within the SiO ${}_{x}$ absorption band. It also shows that the curves with an identical coating thickness deviate within the absorption band depending on the thickness of the substrate. The curves for the coated samples intersect around $1070\;{\text{cm}}^{-1}$ . This intersection changes its position for different angles of incidence or substrate thicknesses. Based on these spectra, the coating thickness cannot be directly determined without prior knowledge of the exact thickness of the substrate.

Interference fringes are not observed in the spectra as metal substrates suppress them increasingly better with approaching a perfect conductor (see the Fundamentals section).

Fewer variations of the reflectance are observed for the uncoated films. The reflectivity at the air/polymer interface is reduced compared to an air/SiO ${}_{x}$ interface. Additionally, in comparison for coated polymers, the incident IR wave is reflected twice: at the interface of air and coating and at the interface of coating and polymer. Consequently, reflectance variations from interferences are more dominant and visible in the spectra of the coated films.

### Implemented Multi-Angle Averaging Approach

By using the multi-angle averaging approach, spatial incoherence is imitated by averaging the intensities of the reflected light at different angles of incidence. The various angles of the incident light cause the path lengths of the light to differ, affecting the interferences and the reflectance.

**Figure 3.**
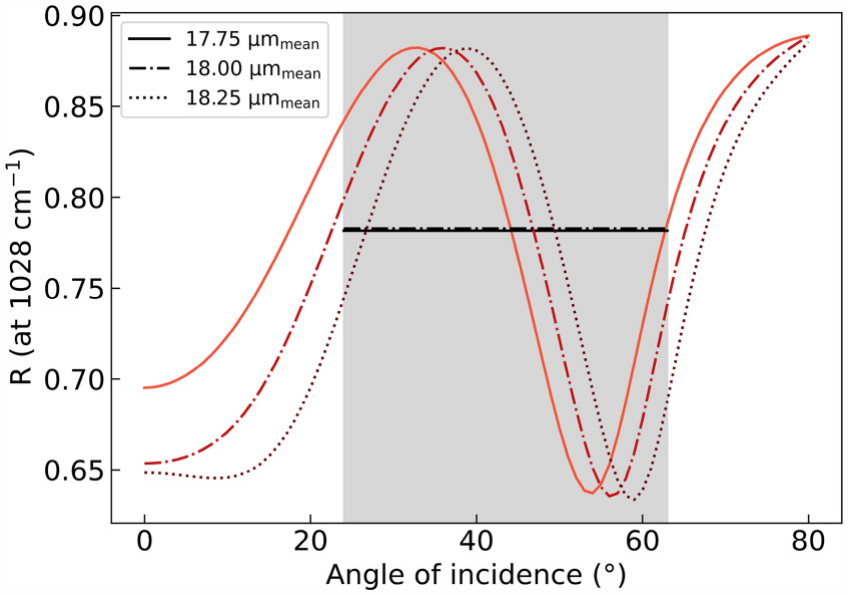
Calculated reflectance over the angle of incidence for substrates with a thickness of 17.75, 18.0, and 18.25 μm . The substrates are coated with 25nm of SiO ${}_{x}$ . The black lines show the arithmetic mean for each thickness over a range of angles of incidences from $0^{\circ }$ to $80^{\circ }$ .

The change in reflectance over the angle of incidence at $1028\;{\text{cm}}^{-1}$ for 25nm SiO ${}_{x}$ coated OPP substrates with a thickness of 17.75, 18.0, and 18.25 μm is shown in Figure 3. The path length of light depends on the refractive index of the layers, the angle of incidence, and the thickness of the substrate, and the curves are calculated with the TMM. The black lines correspond to the arithmetic mean for each thickness of the substrate. The mean values are identical; minor deviations are negligible.

Through averaging over about one period, the interference patterns disappear. For SiO ${}_{x}$ coated OPP polymers with nominal thicknesses of 18μm , calculations indicate that $40^{\circ }$ difference in the angle of incidence results in a consistent average reflectance across slightly varying substrate thicknesses.

The calculated resulting signals based on this approach are shown in Figure 2c. The curves are calculated for the same coatings and substrates as described above; the parameters of the model remain unchanged. Each curve represents the arithmetic mean of the reflectance in the IR spectral range for the incidence angles at $24^{\circ }$ to $63^{\circ }$ with increments of $1^{\circ }$ for each film. Variations in intensity due to fluctuations in the substrate thickness are significantly reduced by a factor of 10. The calculations demonstrate that the coating thickness can be precisely determined on the basis of this calculation model.

## Experimental Method

To validate the multi-angle averaging approach introduced in the Fundamentals section, we need film samples of uncoated and coated polymers. In addition, we also require a setup that measures the intensity of the reflected light over the spectral IR range at different angles of incidence. We will describe the samples, their characteristics, and the measurement setup in the following.

### Samples

The flexible polymer substrates are industrially processed rolls of coated OPP with a thickness of around 18μm . They are coated with SiO ${}_{x}$ by electron beam evaporation. The thickness of the coating is expressed as the surface mass density of silicon, where 1nm approximately corresponds to 1nm of SiO ${}_{x}$ , taking into account the molar mass of silicon, oxygen, and the density of SiO ${}_{x}$ . The samples used here have a surface mass density of 15, 35, 65, and 90 ${\text{mg/m}}^{2}$ , with an accuracy in the range of $\pm 2\;{\text{mg/m}}^{2}$ . These samples were provided as cut pieces and evaluated by Amcor Flexibles Kreuzlingen AG from Switzerland.

**Figure 4.**
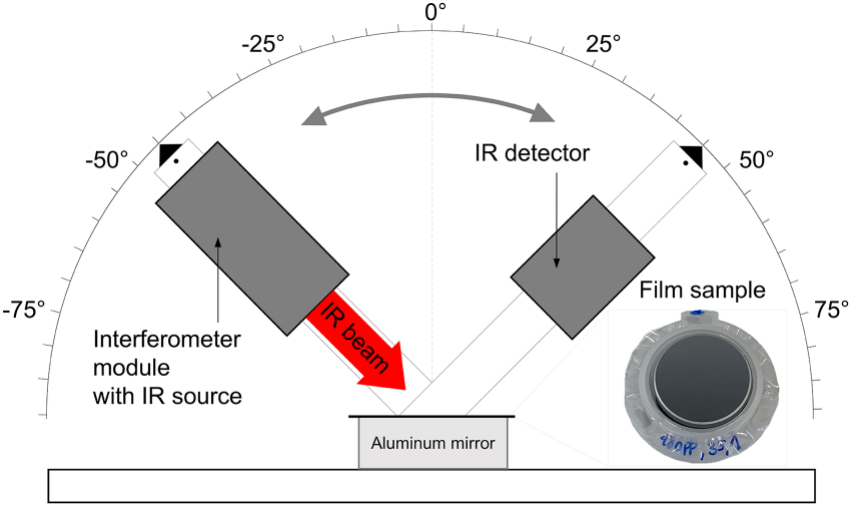
Schematic setup of the IR source and IR detector with variable angle of incidence and a photo of the clamped film sample on a frame placed on an aluminum mirror.

To handle the thin films and to create a flat surface, we clamp the film pieces onto a frame with a diameter of 2.54 cm (1-in.), representing one sample, as shown in the insert of Figure 4. An aluminum mirror with high reflectance is used as background material. Samples with uniform coating thickness are cut from a 20cm×20cm sheet. Over this area, we assume the coating thickness to be homogeneous across the samples.

### Measurement Setup

The previously described samples and the aluminum mirror are placed in the center of the measurement setup, as shown in Figure 4. An IR source and a detector are mounted on rotatable arms, with the axis of rotation positioned at the center. These components are used to measure the intensity of the specular reflection from the samples. Angle markings at the end of the arms, relative to the normal of the sample surface, ensure that the reflected light beam hits the detector. Furthermore, the markings and rotatability of the arms allow for adjusting the angle of incidence, enabling the implementation of the multi-angle averaging approach (see the Fundamentals section). The angle of incidence is adjusted from $24^{\circ }$ to $63^{\circ }$ in increments of $1^{\circ }$ with an accuracy of approximately $\pm 0.2^{\circ }$ .

The FT-IR spectrometer model OEM011 from ARCoptix S.A from Switzerland, consisting of an interferometer module with an IR source and an IR detector, is used for the measurements. It covers a spectral range from 830 to $5000\;{\text{cm}}^{-1}$ , including the SiO ${}_{x}$ absorption band. The diameter of the measuring spot is determined to be 5mm using a variable aperture. For each sample, the measurement spot remains unchanged at the different angles of incidence. All measurements were performed with unpolarized light and a spectral resolution of $4\;{\text{cm}}^{-1}$ . An average of 10 spectra per measurement is taken to optimize the results. The medium Norton–Beer apodization is applied to calculate the spectra.

All measurements are normalized against the spectrum of a bare aluminum mirror to enhance comparability and to deviate the reflectance spectra from it. The intensity of light reflected from the aluminum mirror is considered as the incident light intensity. Offsets of the measured curves caused by the spectrometer are corrected by aligning the curves in a spectral range unaffected by the coating. The measurement time for one sample over all angles of incidence takes around 3 minutes and is performed at room temperature ( $21{\;}^{\circ }\text{C}$ ).

### Determination of the Variation in Substrate Thickness

The variations are measured with the FT-IR spectrometer described above in the spectral range from 1667 to $2500\;{\text{cm}}^{-1}$ . The polymer shows almost no absorption in this range, and the wavelength is large enough to identify variations in the thickness of 1μm . Measurements are normalized against the spectrum of a bare aluminum mirror. We used air as a background to clearly distinguish constructive and destructive interferences. Five uncoated film samples were measured with an angle of incidence set to $45^{\circ }$ .

## Results and Discussion

The measurement results of the substrate thickness confirm its variation, as will be shown below. The measured intensity of the reflected IR light for the samples and the setup described in the Experimental Method section will be shown and will validate the multi-angle approach explained in the Fundamentals section. We conducted the following results at a single incidence angle and multiple angles. Furthermore, we will discuss implementing the approach to an inline measurement system.

### Variation of the Substrate Thickness

**Figure 5.**
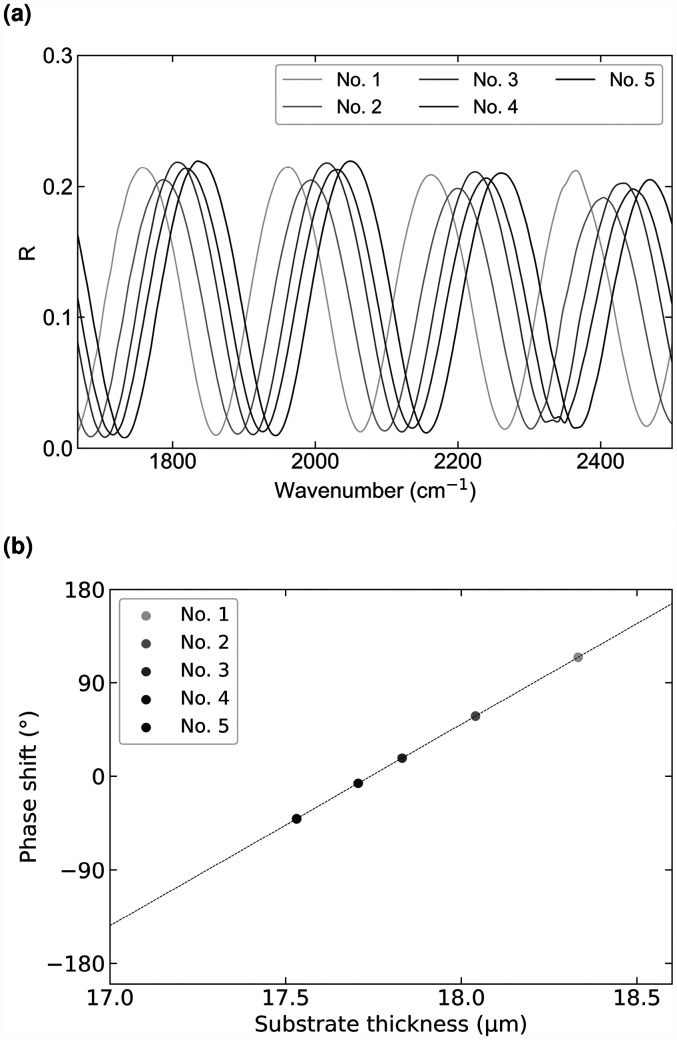
Panel (a) shows the measured reflectance spectra for five uncoated samples with air as background in the range of 1667–2500 ${\text{cm}}^{-1}$ at $45^{\circ }$ angles of incidence. The linear correlation between the phase shift of the oscillation and the substrate thickness is displayed in panel (b), calculated by the TMM. The points on the line mark the phase shift of the measured oscillations.

As shown in Figure 5a, the reflectance of a bare polymer film in the range of 1667–2500 ${\text{cm}}^{-1}$ results in a harmonic oscillation (see the Fundamentals section). Their phases change. We analyze the value of the phase shift using the fast Fourier transformation. Additionally, a theoretical phase shift was calculated using the TMM for known substrate thicknesses, leading to a periodic linear correlation between the substrate thickness and the theoretical phase shift. As shown in Figure 5b, gray line. The measured values (displayed as black and gray dots) were compared with the calculated ones, which led to a thickness of the polymer substrate between 17.5 and 18.5μm . Consequently, the variation in substrate thickness is around ±2.5% for the polymer films used here.

As the phase shift is determined on the basis of a calculation model, the absolute values could slightly differ from the actual values, but the relative variation of the thickness remains unchanged.

### Measurement Results of the Coating Thickness

**Figure 6.**
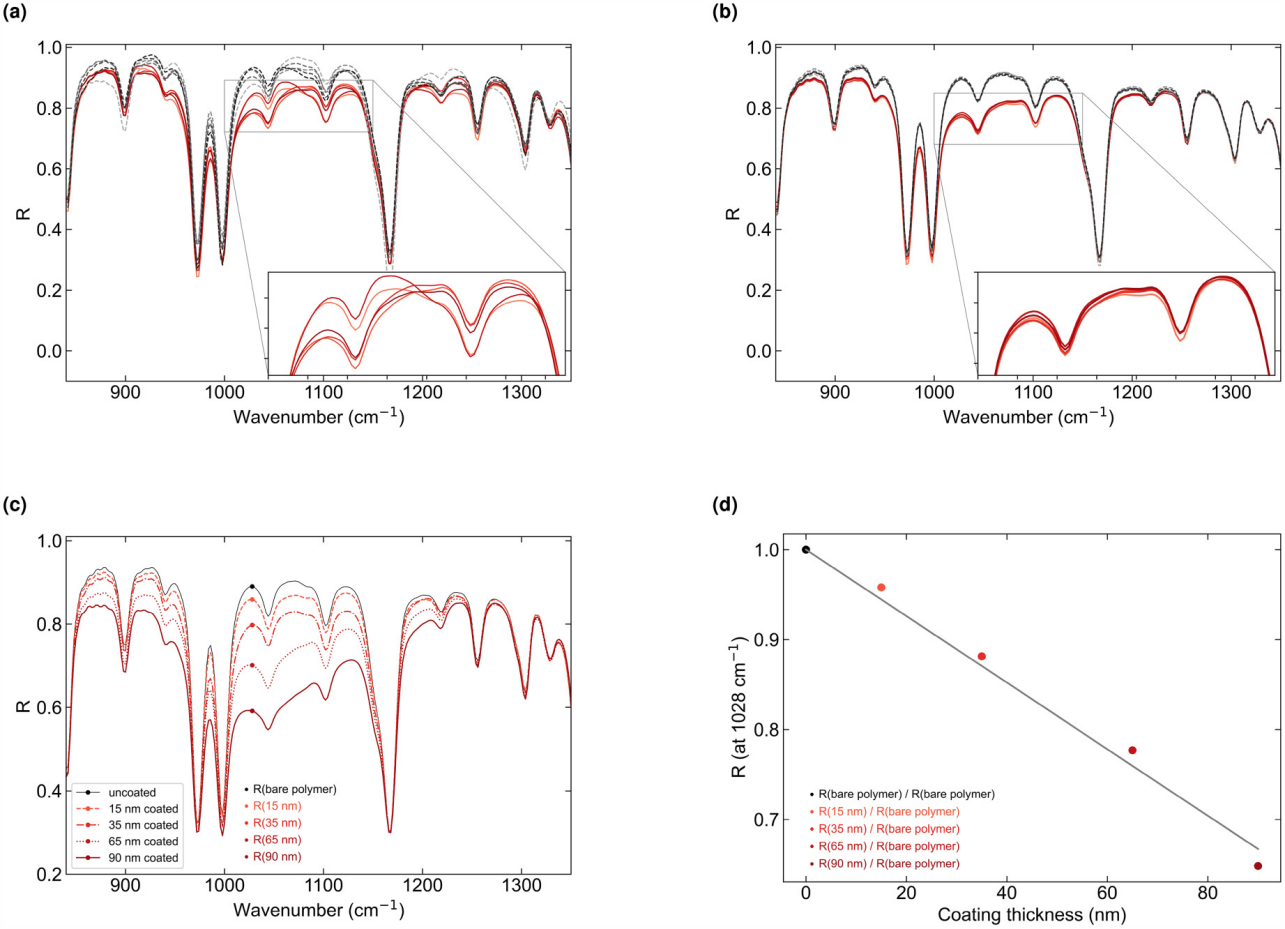
Panels (a) and (b) show the measured reflectance spectra for five uncoated (dashed lines) samples and five coated (solid lines) samples. The coating has a thickness of 35nm . Spectra of panel (a) are measured at $30^{\circ }$ angles of incidence. Panel (b) shows an averaged spectrum for each sample, measured at $24^{\circ }$ to $63^{\circ }$ angles of incidence with $1^{\circ }$ increments. Panels (c) and (d) show measurement results for one uncoated and four coated samples of different coating thicknesses. The coating layers have a thickness of 15, 35, 65, and 90 nm. Spectra of panel (c) are measured with the multi-angle averaging approach. Panel (d) shows the derived reflectance and regression line at $1028\;{\text{cm}}^{-1}$ wavenumbers.

In Figure 6a, we show reflectance spectra in the range of 840–1350 ${\text{cm}}^{-1}$ wavenumbers, at a fixed angle of incidence of $30^{\circ }$ , for five uncoated samples (dashed lines) and five coated ones (solid curves) with a thickness of 35nm .

The pronounced absorption peaks, for example, observed at 970 and $998\;{\text{cm}}^{-1}$ , are attributed to the polymer substrate itself.^23^ As expected, the reflectance of the coated sample is reduced in the range of the SiO ${}_{x}$ absorption band (see the Fundamentals section) compared to the reflectance of the uncoated samples. Furthermore, the measured curves are qualitatively in agreement with the simulated ones, shown in Figure 2b. Nevertheless, the measured curves of the coated samples vary by up to 10%. The results prove that the thickness of a sample at a single measurement spot cannot be precisely determined.

Furthermore, if the thickness of the polymer substrate is unknown, some curves of the coated samples show closer alignment than others. This trend is also evident in the measured spectra at other angles of incidence, suggesting that the substrate thickness of specific samples is similar to that of others.

In Figure 6b, we show the averaged spectra for angles of incidence between $24^{\circ }$ and $63^{\circ }$ with increments of $1^{\circ }$ for the same samples used in Figure 6a. The measured curves of all coated samples vary only by around 1%, with a similar variation observed for the uncoated samples, proving the benefits of our multi-angle approach as predicted by calculations (see Figure 2c). As a result, it allows for precise determination of the thickness of the coating, in the order of ±2nm , at a single measuring point on thin-coated polymer films.

Figure 6c shows the averaged spectra, measured with the multi-angle averaging approach, for one uncoated sample and each one with a coating of 15, 35, 65, and 90 nm thickness. The resulting curves exhibit a clear difference between the different surface mass densities. From these curves, we derive the reflectance at $1028\;{\text{cm}}^{-1}$ at which we reach the best linear fit.

The resulting correlation between the reflectance R and the thickness is shown in Figure 6d. The reflectance values are normalized to the arithmetic mean of the reflectance of a bare (uncoated) polymer sample at $1028\;{\text{cm}}^{-1}$ , measured at the specified angles of incidence. We conclude that the resulting linear regression line follows the Beer–Lambert approximation^14,24^ in the given thickness range.

Based on the line, we determined the thickness with an accuracy better than ±5nm . Furthermore, the regression line can be utilized to calibrate the measurement setup and determine unknown coating thicknesses. However, as described in the Experimental Method section, the surface mass density was provided by the manufacturer of the film samples. As we assume that this value can also deviate slightly from the actual thickness, it influences our accuracy specification.

### Approaches for an Inline Measurement System

To determine the coating thickness at a single measuring spot, we took 40 measurements at different angles of incidence. These many measurement points are not feasible, especially for inline measurements in R2R coating systems, where rapid measurements are required. In the following, we will discuss implementing the multi-angle averaging approach to a sensor. The sensor should meet the requirements of the Introduction section.

As mentioned in the Fundamentals section, we developed a compact and versatile sensor comprising a thermal IR emitter and a pyroelectric detector that measures the coating thickness of SiO ${}_{x}$ in the nanometer range. The sensor measures the intensity of the reflected light without spectral resolution but measures it over a spectral range covering the absorption band of the oxide. However, in our previous studies, thick polymer substrates were investigated in contrast to the thin films described here. In the following, we describe how our sensor^11,25^ can be adapted to implement the multi-angle averaging approach and to measure thin polymer substrates.

An option is the use of optical lenses. The IR source emits divergent rays at a specific aperture angle, and by utilizing optical components such as concave lenses, its output can be adjusted to achieve the desired divergence. A convex lens placed before the polymer film focuses the light onto it. The focus on the film causes the light to come in at different angles of incidence. The distribution of the rays over the angle of incidence depends on the focal length of the lens. For a sufficiently large distribution ( $40^{\circ }$ ), a small focal length (in the lower double-digit millimeter range of 10–30 mm and a large diameter of the lens (2.54–5.08 cm; 1–2-in.) are required. A second lens behind the sample focuses the rays on the detector again.

However, the intensities of the divergent rays from the source are not homogeneously distributed. The edge rays are less powerful, and their contribution to the resulting reflectance is reduced. This may result in interferences not being fully suppressed, even when measured at different angles of incidence. To overcome this effect, an extended light source consisting of several IR emitters can be beneficial.

The implementation and publication of this setup will be subject to a future study by taking into account the requirements in the Introduction section.

We can already state that the sensor based on the described designs with an implemented multi-angle averaging approach, will be non-destructive, scalable, and compact. In addition, the sensor applies to different polymer substrates, especially those with low absorption (e.g., PE, polyvinyl chloride, or technical polymers such as Zeonor).

The thickness of oxide coatings with an IR region absorption band is measurable. Adapted optical filters can reach absorption bands at spectral positions in the range of around 1250–750 ${\text{cm}}^{-1}$ . Other oxides, such as aluminum oxide, titanium oxide, or nitrides, such as silicon nitride, are common alternative coating materials.

Rapid measurements in R2R coating systems still need to be tested. In future measurements, the metal rolls of the system can provide a highly reflective background, ensuring that the film remains flat and experiences minimal vibrations.

## Conclusion

We show how the thickness of nanometer-thin oxide films can be measured in the IR spectral range on thin and weakly absorbing polymer substrates. For such films, we demonstrate that reflections occur from the interface between the substrate and the medium of the background, leading to interferences. As industrial films vary in thickness by around 5%, the actual measurement signal of the coating thickness is disturbed. We have developed and applied a multi-angle measurement approach that averages out the signal variation due to interference and enables the precise coating thickness measurement at one measurement point. The newly developed method is based on reflection measurements. Measurements are taken at typically used SiO ${}_{x}$ coatings.

In conclusion, our results, measured on industrially coated polymer films, show that the approach can measure film thicknesses of at least 15–95 nm with a precision of ±1nm and an accuracy of ±5nm .

The approach improves precision by a factor of 10 compared to measurements without the approach. The achieved order of magnitude of precision and accuracy is comparable to measurement systems presented in the state-of-the-art. On the basis of calculations, we have also shown that semi-infinite exit mediums of high reflectance improve measurement results.

Measurement of the coating thickness of R2R-produced polymer films is crucial to ensure product quality in mass production. The outcomes and integration potential of this approach could allow for inline quality control of coating thickness and associated product properties on thin, transparent polymer films. Inline control not only facilitates process regulation but could also help to conserve resources in the future. This approach is anticipated to be scalable, versatile, and non-destructive. Further investigation is needed to integrate and test it within a sensor system for final evaluations of measurement results in the application.
